# Assessment of Musculoskeletal Injuries from Domestic Violence in the Emergency Department

**DOI:** 10.1155/2015/721528

**Published:** 2015-05-07

**Authors:** Georgios F. Giannakopoulos, Udo J. L. Reijnders

**Affiliations:** ^1^Department of Trauma Surgery, VU University Medical Centre, P.O. Box 7057, 1007 MB Amsterdam, Netherlands; ^2^Department of Forensic Medicine, Public Health Service, P.O. Box 2200, 1000 CE Amsterdam, Netherlands

## Abstract

Domestic violence is one of the most common causes of nonfatal injury in women, with musculoskeletal injuries representing the second most prevalent manifestation of this form of violence. It is therefore of great importance that healthcare providers such as emergency department (ED) physicians and surgeons are able to recognize and assess these kinds of injuries. In this case report, a woman is described visiting an ED with injuries caused by a fall. Thanks to the knowledge and attention of the ED physician, the real cause of the injury was discovered. What appeared to be an unsuspicious accident was actually the result of intimate partner violence.

## 1. Introduction

Domestic violence is a serious public health and medical problem that is prevalent in all classes of our population. In Netherlands each year over 200.000 women are victims of domestic violence in one form or another [1]. An estimated 12% of the acute injuries of women are caused by domestic violence [2]. More than half of these female victims who are treated for these injuries visit an emergency department (ED) [3]. Three-quarters of the attending physicians would not recognize these cases as abuse related [4].

## 2. Case Description

A 47-year-old woman visiting the ED is complaining of heavy pains in her left forearm. She claims to have fallen against a wardrobe. Examination by the attending ED physician reveals a purple-blue discoloration of the left forearm. He also notices a yellow discoloration around the left eye and forehead. This discoloration possibly dates from an earlier time. X-rays show a fracture of the forearm (both radius and ulna). When the physician expresses his doubts about the cause of the accident, the woman then admits being beaten and abused by her husband frequently. The fracture of her forearm was caused by a blow from a baseball bat and the “black eye” by a punch to the face at an earlier time. Further physical examination reveals extensive fingertip bruising caused by hefty grasping of the arms, back and buttocks, outline hematomas caused by beating with a round and cylindrically formed object (wooden spoon), and hematomas caused by beating with a looped cord.

## 3. Discussion 

Previous studies have shown that musculoskeletal injuries represent the second most prevalent manifestation of intimate partner violence [5, 6]. It can be assumed that most ED physicians and (orthopedic) surgeons are treating victims of family violence, knowingly or unknowingly, in their practices [7–9].

In this case we observe a conscientious ED physician. Nevertheless even with visible exterior injuries physicians often overlook the possibility of abuse related causes. This is a serious matter because in Netherlands alone each year more than 80 women die as a direct consequence of domestic violence, whereas early recognition might have avoided the fatal outcome in most cases [1]. This situation has not been changed for the last decade [10, 11].

Reasons for not recognizing the abuse by physicians are often explained by lack of education and training, playing down the severity of the injury, fear of being mistaken, not wanting to be the policeman, not knowing the proper referral agencies, and lack of time. Bhandari et al. concluded from their study, in which 186 orthopedic surgeons participated by responding to a questionnaire, that discomfort with the issue and lack of education have led to misconceptions among Canadian orthopedic surgeons about intimate partner violence [5].

It is also commonly known that many women (>60%) are reluctant to speak of the violence because of shame and fear and do not dare speak of the true nature of their injuries [12, 13]. It is therefore the reason that the “fall from the stairs” or the “fall against the wardrobe” is often used as the reason for the injury [14]. More than half of these women would offer honest answers to the questions if the physician had only asked them directly. This is an important factor for opening the problem for discussion which stops the violence in 50% of the cases [15]. Another research has shown that in 72%–93% of the patients questioned by the physician over domestic violence where there was none in question those people reacted positively, expressing words of understanding, conscientious behavior, and agreeing it was advisable to subject victims to this line of questioning [16, 17].

When assessing injuries, one should always question whether the cause was accidental or intentional. Many accident injuries are caused by a fall, jolt, or jab. Preferential spots are the forehead, chin, knees and elbows, the inner side of hands and shins, and areas where bone structure exists low beneath the skin as in spinous processes and crista iliaca. Accidental injuries can also be caused by improper use of sharp utensils and machines. One-third of the injuries are to the more uncommon spots such as the eye, side of the face, throat and neck, upper arms and upper legs, mouth, outer side of the hand, back, hair on head, shoulder and chest, genitals, and buttocks [18]. Injuries and irregularities in these spots should be treated with more than the usual curiosity and necessary investigation. The physician should ask himself if the story matches with what is being observed. In regular abuse cases, one often observes contusions in various stages of healing (red, blue, green, yellow, and brown). They are often found in spots which are not easily accessible to the victim or there is question of various contusions of the same form.

But also with other injuries such as scrapes, tears, cuts, burns, injuries to the central nervous system, fractures, and bite wounds, one should consider the fact of it having been caused by someone else.

One must realize that for the outside world the only injuries that are visible are those not covered by clothing as in the face and the outer side of the hand. There might also be the injuries under the clothing that are not immediately visible. Research showed that 80% of the injuries were visible and 20% were hidden by clothing. But in 85% of the victims, there were injuries found in visible spots as well as those made invisible by clothing. This is because in 60% of the cases of victims of injuries the cause is usually the result of various forms of infliction to various parts of the body [18].

Physicians should familiarize themselves with recognizing the signs of injuries. When there is a question of abuse, the whole body should be inspected and it should be noted in the differential diagnosis. A high index of suspicion is necessary and the knowledge can be acquired by studying photo material of many forms of visible injuries [19]. Furthermore, it was demonstrated that after a brief training domestic violence was recognized four times more often than those who had not had the training [20].

If there is question of domestic violence, the ED physician can advise consultation with the GP. They can also make referrals to an advice and support center for domestic violence or a battered-women-house. Advising the victim to report the case to the police is also an option to be considered.

## 4. Conclusion

Knowledge and early recognition of inflicted injuries of domestic violence should be improved among healthcare providers such as (orthopedic) surgeons, ED physicians, and GPs. Domestic violence remains a difficult subject to discuss for the victim as well as the physician. Nevertheless, discussing the matter is the only way to make strides with this problem. This can help victims out of their isolation so they can be adequately helped and can also save lives for some. If physicians are not confident in critically examining injuries, they should ask professionals like forensic physicians for help. One must realize that ignoring the problem is even more harmful.
